# A case study on anthrax in an eight-month old infant at Kyrgyz Republic

**DOI:** 10.6026/973206300200301

**Published:** 2024-03-31

**Authors:** Mamatkulova Nazgul Mamatkulovna, Zholdoshev Saparbai Tezekbaevich, Utepbergenova Gulmira Alkenovna

**Affiliations:** 1 Department of public health, International Medical Faculty, Osh State University, Osh City, Kyrgyzstan; 2 Department of Infectious Diseases, International Medical Faculty Osh State University, Osh City, Kyrgyzstan; 3 Department of Infectious Diseases and Physiology, International Kazakh-Turkish University named after H.A. Yasawi, Kazakhstan

**Keywords:** Anthrax, Kyrgyz Republic, child, epidemiological history, clinical case, vaccination, clinical course, focus of anthrax

## Abstract

Anthrax remains a threat, especially in countries like Kyrgyzstan with developed livestock farming. Despite preventive efforts, sporadic outbreaks endure on an annual basis, transmitted from infected animals to humans. Here, we report a severe anthrax case in an 8-month-old child known to be caused when a sick calf was slaughtered in the neighborhood without proper protocols, resulting in intra-family infection. This underscores the importance of swift diagnosis, treatment, preventive measures, and awareness of zoonotic infections, animal vaccination and adherence to sanitary and veterinary protocols.

## Background:

Anthrax, a rapidly spreading infectious illness impacting both animals and humans, commonly presents symptoms such as skin lesions, fever, and intoxication. Animals acquire it through exposure to contaminated soil or water, or by ingesting tainted food [1]. Humans mainly get anthrax from sick or dead animals, or through products derived from them, like meat, leather, wool, and bones obtained during unauthorized slaughter of infected animals [2]. Anthrax spreads to humans mainly through contact with infected animals, their products, or contaminated soil. WHO reports 2,000 to 20,000 cases annually worldwide? It's common in countries with intensive livestock farming, such as Kyrgyzstan in Central Asia [3]. The persistence of numerous untracked soil sources of anthrax allows the pathogen to remain viable in the environment, leading to sporadic outbreaks among humans and animals [4]. Recent data from the Republican Center for Quarantine and Especially Dangerous Infections shows a consistent rise in anthrax cases in the republic, ranging from 2 to 73 cases of human infection cases [5].

Osh and Jalal-Abad regions report the highest number of cases; Chui region sees sporadic cases, while anthrax incidence in the south is complicated by unsanitary soil foci [6]. Out of 1,200+ soil anthrax foci, 555 (44.9%) were on ground, with 466 (83.9%) fenced; in Osh and Jalal-Abad, 314 (51.6%) were on ground, 83% fenced and concreted . Over a decade, most cases from 2013-2023 occurred in Jalal-Abad (45.5%) followed by Osh (27.2%), Talas had 11.6%, Naryn 7.4%, Chui 4.7%, and Batken 3.4% respectively. Also, Issyk-Kul region had no cases. Osh region's 7 districts include Alai, Aravan, Kara-Suu, Kara-Kuldzha, Chon-Alai, Nookat, and Uzgen, with a 2023 population of 1,460,400. In Osh, Kara-Kulzha (40%) and Kara-Suu (35%) districts had the highest incidence over the decade [8]. The Kyrgyz Republic, with 6.6 million people, faces endemic anthrax, particularly in its rural south where herding is common and the cases have steadily increased over 5 years, with spikes in 2015, 2018, and 2020 [9]. In recent years, the southern region has seen annual cases of cutaneous anthrax, peaking at 27.5% in 2018, with 22 cases in 2015 (intensity indicator: 20.1) and a worrisome 18.3-fold increase to 5 cases in 2020 (intensity indicator: 4.8) [10]. Hence, we report a case for anthrax in an eight-month-old infant from the Kyrgyz Republic towards revised guidelines for healthcare professionals aimed at the prevention and treatment of anthrax, while also aiding emergency planners in strategizing for potential aerosol releases of *B. anthracis*.

## Materials and Method:

The primary sources of information regarding the child's health status included data extracted from the outpatient card, developmental history records (Form 112/Y), comprehensive medical records spanning the entire duration of inpatient treatment, and interviews conducted with the child's mother. Additionally, findings from epidemiological investigations and veterinary examinations of the infection site were incorporated into the assessment process.

## Description:

On the evening of August 5, 2023, a child exhibiting symptoms suggestive of anthrax was admitted to the pediatric infectious diseases ward of Osh City Clinical Hospital. According to the child's mother, over the preceding 48 hours, there had been intermittent spikes in body temperature up to 38 degrees Celsius, accompanied by disruptions in sleep patterns, overall fatigue, heightened anxiety, and the emergence of a rash resembling a reddish patch on the lower right cheek, which gradually expanded in size. The child, the eldest in the family, was born at full term on December 7, 2022, following an uneventful pregnancy. The mother affirms that the child's growth and development align with age expectations. Routine vaccinations have been administered in accordance with the national immunization schedule. Exclusive breastfeeding was practiced until the age of 6 months, after which complementary foods were introduced. Notably, the child has a history of frequent illnesses and is under the care of a neurologist for encephalopathy and a cardiologist for a congenital atrial septal defect (Q21.1). Additionally, there is a documented allergic history exacerbating the child's health concerns.

Upon admission to the hospital, the child presented with a severe condition characterized by a fever of up to 38 degrees Celsius and signs of intoxication such as anxiety and loss of appetite. Physical examination revealed a height of 72cm and a weight of 9.7kg. The child maintained clear consciousness with no signs of meningeal involvement. A localized examination revealed a vesicle filled with serous fluid, measuring 0.5 x 1 mm, devoid of a black scab, surrounded by a red cushion on the lower corner of the right cheek. Hyperemia and significant swelling were observed at the site of the vesicle formation. Despite these manifestations, the affected area of the face was non-tender upon palpation. There were no notable enlargements in the submandibular and cervical lymph nodes. Oral mucosa appeared clean, although the pharynx exhibited hyperemia without plaque formation. Nasal breathing remained unobstructed. Chest examination revealed a cylindrical shape with symmetrical participation in the respiratory process and no retractions of the lower sternum. However, breath sounds in the lungs were noted to be harsh, with a respiratory rate of 44 breaths per minute and a pulse oximetry saturation of 96%. Abdominal palpation yielded a soft and painless abdomen, with a large protruding umbilical ring. Neither the liver nor the spleen was palpable, and regular stool passage was noted.

In order to confirm the diagnosis and establish appropriate medical strategies, a series of laboratory and instrumental examinations were conducted. The results of these tests are outlined in Table 1. The treatment administered to the child adhered to the guidelines outlined in the Ministry of Health of the Kyrgyz Republic's Order No. 1446, issued on December 14, 2022. This treatment protocol encompassed practices such as free breastfeeding, maintaining a fluid balance of 100 ml/kg per day, and administering antibacterial therapy, including four doses of intravenous ampicillin daily and two doses of intravenous Ciprox administered via drip.

On July 31, 2023, the parents and uncle of the child were admitted to the infectious diseases department of Osh City Clinical Hospital due to suspected anthrax. Subsequent laboratory tests confirmed the diagnosis. Epidemiologists, in collaboration with veterinarians, initiated an investigation into the initial outbreak. According to the child's grandfather, the family is involved in livestock farming in the village. In mid-July, they spontaneously slaughtered a sick 2-month-old calf. The child's father, mother, and uncle were involved in the slaughter process. The mother had contact during the handling of internal organs and the gastrointestinal tract. The calf's meat was then divided and used for cooking. Three days later, on July 27, 2023, all three family members experienced a simultaneous increase in body temperature. This was followed by the appearance of a spot resembling a small mosquito bite, which developed into vesicles filled with serous fluid. By the fifth day, ulcers appeared in various locations: the mother's chin, the uncle's shin, and papules and pustules on the father's forearms and fingers. Consequently, they were promptly hospitalized. The child, who began complementary feeding at six months, had been fed pre-chewed food made from contaminated meat. Additionally, the child was in close contact with the mother. The child's relatives took her to the local clinic on August 5, 2023, due to symptoms including increased body temperature, sleep disturbances, decreased appetite, anxiety, and a facial rash.

At home, the child was administered antibiotics and antipyretic syrups, but they showed no effect. Due to the severity of the condition, the child was admitted to the hospital. Subsequent epidemiological investigation revealed that in mid-July 2023, the owner had immunized all his livestock against anthrax. This vaccination procedure was carried out by a local private veterinarian using the Antravac vaccine, a live vaccine manufactured by Agrovet LLC, Russian Federation, designed to combat animal anthrax (strain 55-VNIIVViM). The recommended vaccination schedule involves administering the vaccine to small and large cattle at 3 months of age, with foals receiving it at 9 months. Booster shots are given every 6 months, followed by annual revaccination for cattle. However, despite these guidelines, the veterinarian inoculated a 2-month-old calf against anthrax. Tragically, a week later, the calf perished, likely due to either complications arising post-vaccination or succumbing to anthrax despite vaccination. Upon inspecting the veterinarian's thermal container, it was discovered that the cold chain had been compromised, with temperatures during vaccine transportation (optimal range: 2-8°C) not maintained. In ambient temperatures of +27°C, the Antravac vaccine, intended for anthrax prevention in farm animals, was stored in a thermal container lacking a cooling element and temperature recorder to ensure adherence to the required temperature regime.

In the initial area of infection, soil was sampled from the location where a calf was slaughtered (as per Examination Protocol No. 101-102 dated 08/02/2023) to detect the presence of the anthrax-causing agent. Analysis results from 08/04/23 confirmed the presence of Bacillus anthracis in the soil. Veterinary authorities delineated the boundaries of the infection source area, posing risks of disease transmission to both animals and humans, and implemented necessary sanitary and anti-epizootic measures. Immediate antibiotic prophylaxis was administered to relatives and individuals in direct contact with the deceased sick animal or those who consumed its meat. The community remains under vigilant medical surveillance. The child was discharged on August 15, 2023, in satisfactory condition, with improved appetite, restful sleep, and clear, pale skin. The vesicle on the face had completely disappeared. Continued breastfeeding was advised, along with on-going monitoring by a family general practitioner, neurologist, and cardiologist. The mother received training to identify symptoms of severe childhood illnesses, guidance on childcare and feeding practices, and a discussion on the significance of preventive vaccinations.

The primary mode of anthrax transmission occurs when individuals handle the carcass of a dead or forcibly slaughtered sick animal, resulting in significant pathogen exposure through blood, intestinal contents, and similar means. Literature does not document instances of disease transmission from individuals with cutaneous anthrax to healthy individuals, nor has there been any reliable evidence of person-to-person transmission, even with close contact. Despite extensive scientific research spanning many years, no clinical cases of anthrax infection in infants have been reported in the past decade. While clinical characteristics of anthrax in children have been detailed in Afghanistan, the documented age range for affected children was between 6 and 16 years old.

## Conclusion:

Anthrax, a potentially fatal disease, can strike individuals of any age, including vulnerable infants. This underscores the critical need for prompt diagnosis, hospital care, and effective treatment to ensure favourable outcomes. However, the impact of anthrax extends beyond individual cases; it poses significant challenges, particularly in low and middle-income countries. The lack of adequate healthcare infrastructure and awareness among rural communities heightens the risk of zoonotic infections. Farmers who rely on livestock for their livelihoods may inadvertently expose themselves and their families to the disease due to insufficient understanding of preventive measures. Enhanced research efforts, focused on low and middle-income countries, are essential to understand the disease better and develop sustainable solutions. Through collaboration and commitment, we can strive toward a world where anthrax no longer poses a threat to public health.

## Figures and Tables

**Table 1:** Comprehensive compilation of pathological tests and associated findings

**Laboratory and Instrumental Studies**	**Results**
1.Bacteriological Examination of Ulcer for Anthrax (06.08.23)	Bacillus anthracis detected
2.Bacteriological Blood Test for Anthrax (06.08.23)	Bacillus anthracis not detected
3.General Blood Test (06.08.23)	Hemoglobin: 100g/l Red Blood Cells: 3.6x10^12/l Color Index: 0.83 White Blood Cells: 13.2x10^9/l ESR: 26 mm/h Segmented: 14x10^9/l Lymphocytes: 78.5x10^9/l Monocytes: 7.8x10^9/l
4.General Urine Analysis (06.08.23)	Volume: 10.0 Colour: Yellow Specific Gravity: 1025 Protein: 0 Glucose: 0 Leukocytes: 2-3/field of view Squamous Epithelium: 4-11/field of view Salt: Urates
5.Scatological Research (07.08.23)	No changes
6.Liver Tests (07.08.23)	Total Protein: 61.4 g/l Thymol Test: 2.7 units Total Bilirubin: 16.9 µmol/l Indirect Bilirubin: 15.6 µmol/l Direct Bilirubin: 1.3 µmol/l B-lipoproteins: 3900 mg/l ALT: 0.14 mmol/l
7.Kidney Function Tests (07.08.23)	Residual Nitrogen: 0.16 g/l Urea: 2.77 µmol/l Creatinine: 49 µmol/l
8.Rheumotests (07.08.23)	Total Protein: 61.4 g/l C-reactive Protein: 6 mg/l RF: 8 units/L ASO: 200 units/L
9.X-ray of Chest in Direct Projection (09.08.23)	No infiltrative changes expanded heart boundaries
